# Activity of frontal pole cortex reflecting hedonic tone of food and drink: fNIRS study in humans

**DOI:** 10.1038/s41598-018-34690-3

**Published:** 2018-11-01

**Authors:** Yuji Minematsu, Kayoko Ueji, Takashi Yamamoto

**Affiliations:** 1 Health Science Research Center, Kio University, 4-2-4 Umami-naka, Koryo, Kitakatsuragi, Nara 635-0832 Japan; 2 Department of Nutrition, Faculty of Health Sciences, Kio University, 4-2-4 Umami-naka, Koryo, Kitakatsuragi, Nara 635-0832 Japan

## Abstract

Cognitive and hedonic aspects of taste have been studied using different neuroimaging techniques in humans. However, the methods used are unsuitable for easy monitoring of hedonics induced by intake of foods and beverages. Here we have tried to monitor changes in oxygenated hemoglobin (oxyHb) levels in the anterior prefrontal cortex (aPFC, frontopolar cortex, Brodmann area 10) in response to intake of hedonically different edibles in healthy adults. When subjects tasted sweet and bitter solutions freely without any particular instruction, cortical activation varied greatly among subjects and between the two stimuli, and no consistent results were obtained. Subjects then ate or drank preferred (hedonically positive) and disliked (hedonically negative) edibles. Although these stimuli differed among subjects, hedonically positive stimuli decreased oxyHb, whereas hedonically negative stimuli increased oxyHb, particularly in the ventral aPFC. When subjects tasted 4 kinds of jellies with different flavors and evaluated the degree of pleasantness, oxyHb level in the ventral region correlated negatively with pleasantness score. These results revealed that pleasant and unpleasant edibles tended to elicit decreased and increased oxyHb levels, respectively, within the ventral aPFC, suggesting that monitoring of oxyHb in this region may prove useful for objective evaluation of pleasantness of food and drink.

## Introduction

Food companies evaluate the palatability and acceptability of new foods and beverages mainly using a variety of sensory tests. Food scientists and consumers also evaluate the pleasantness of food and drink using sensory tests. Although such sensory tests are very common and useful, the scores tend to vary according to the personal opinions of the judges and factors including internal and external conditions. Utilization of more objective methods has long been desired for hedonic evaluations of foods and drinks. Since the evaluation of palatability (or pleasantness) is processed in the brain, monitoring of brain activity is expected to offer a candidate tool for such objective judgment. A number of studies have been published on the location and magnitude of brain activity in response to the taste and flavor of different edibles with positive and negative hedonics. Through such studies, qualitative and quantitative aspects of taste are generally accepted to be processed in the operculum and insula, representing the primary cortical gustatory area^1–5^, while food reward value and subjective pleasantness are processed in the orbitofrontal cortex (OFC), as the secondary cortical gustatory area^6–9^, along with the lateral prefrontal cortex (LPFC)^10–14^, anterior cingulate cortex (ACC), amygdala, reward system and hypothalamus^7^.

Most of these studies have been performed using neuroimaging techniques such as functional magnetic resonance imaging (fMRI), positron emission tomography (PET) and magnetoencephalography (MEG). These methodologies allow accurate localization of brain activities in response to taste stimulation, but show limitations in the need for holding the head strictly stationary, which prevents subjects from more natural and free tasting or ingestion of food and drink. The present study therefore used functional near-infrared spectroscopy (fNIRS) to explore cortical areas involved in hedonic evaluations. This technique allows the subject to eat and drink without severe restrictions, because the subject wears only a set of optodes consisting of emitters and detectors of near-infrared light. As an optical method is based on the properties of hemoglobin in absorbing near-infrared light^15,16^, fNIRS can only measure cortical activity, and is unable to measure the activity of deeper areas, such as the primary and secondary gustatory areas or the cingulate cortex and amygdala.

Our pilot study tried to measure activity from the LPFC, since recent studies have suggested that the LPFC is a crucial area for cognitive processing of taste and other food-related behaviors^17^, the dorsolateral prefrontal cortex (DLPFC) is involved in memory formation in association with taste^12^ and the ventrolateral prefrontal cortex (VLPFC) is activated by perception of taste^10,11,14^. In addition to the lateral part of the prefrontal cortex, we placed a set of optodes on the forehead for the anterior (frontopolar) prefrontal cortex (aPFC, Brodmann area 10), since the forehead allows very easy placement of optodes due to the lack of underlying hair and muscles. Sato *et al*.^18^ reported that measurement of extracranial hemodynamics with optodes on the temporal region of the head exhibited a very good correlation with saliva secretion in response to taste stimulation. They also suggested that the marked extracranial hemodynamic changes are mainly related to arteries around the parotid gland. Since the position of a set of optodes for fNIRS measurement of the DLPFC and VLPFC is essentially the same as that used in this report, careful consideration should be given to discriminating between intra- and extracranial hemodynamic changes. We have therefore discarded recordings from the temporal regions. On the other hand, we noticed small but significant hemodynamic changes in the aPFC during stimulation with different tastes and edibles. This area, of course, is not directly concerned with processing taste information, but is related to a cognitive system that facilitates either behavior required to concentrate on the current sensory input or the mental processing that accompanies self-generated thoughts^19^. Although the precise functions of the aPFC still need to be elucidated, the present study explored the possible representation of affective reactions related to hedonic evaluations of food and drink.

## Results

### Stimulation with sweet and bitter solutions (Experiment 1)

**Figure 1:**
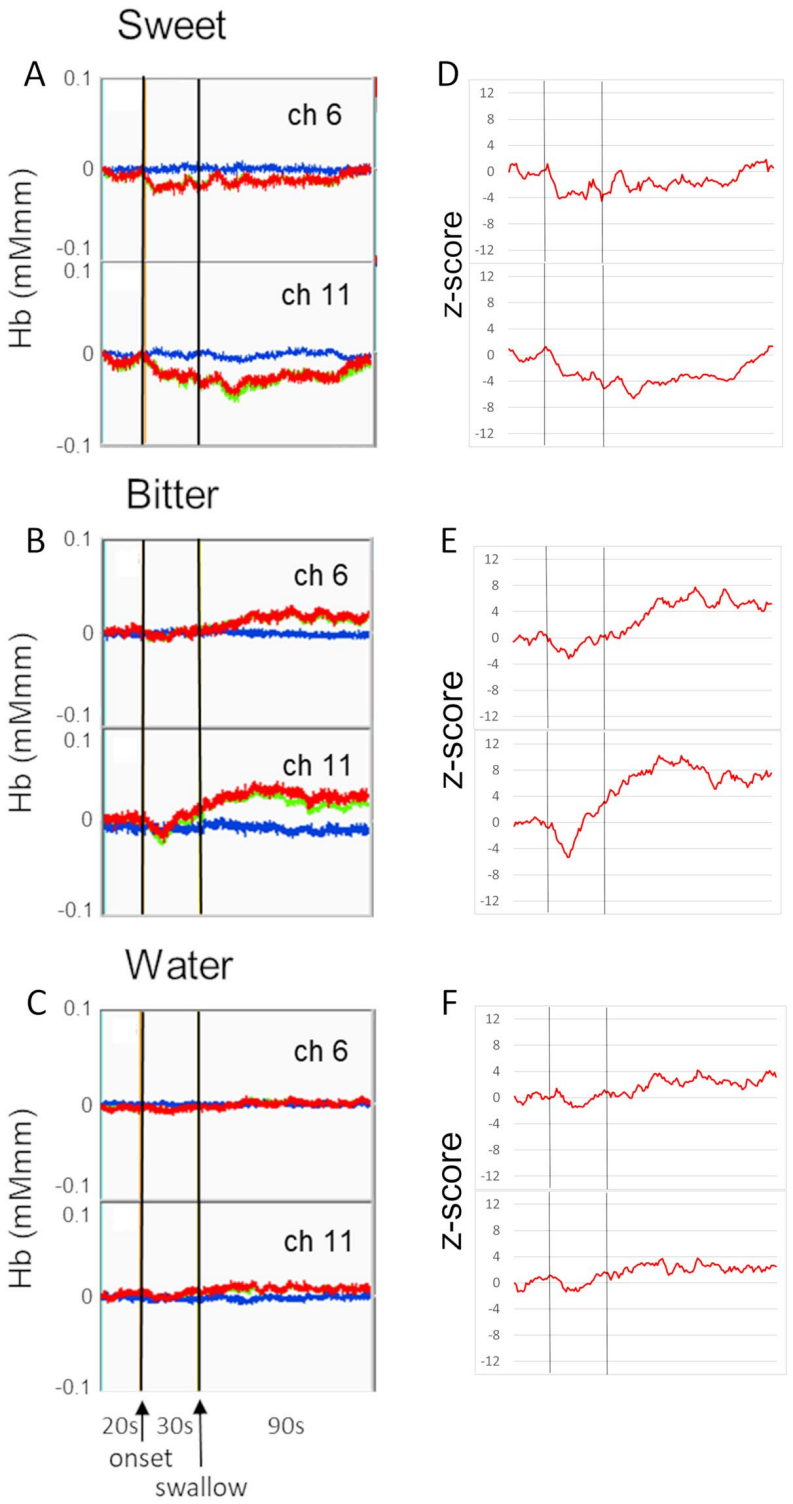
Sample records of cerebral hemodynamics (**A–C**) and corresponding time course of z-scores (**D–F**). Responses were obtained from chs 6 and 11 in response to sucrose (sweet), SOA (bitter) and water in one subject. Sweet stimulation decreased the oxyHb concentration, while bitter stimulation increased the oxyHb. The first vertical line, onset of stimulation; the second vertical line, swallowing; red trace, oxyHb level; blue trace, deoxyHb level; green trace, totalHb.

Figure 1 shows sample raw records and corresponding time series of z-score in two channels (ch 6 and ch 11) to sweet (sucrose), bitter (sucrose octaacetate, SOA) and water stimulations in a subject. Oxygenated hemoglobin (oxyHb) level decreased gradually after the onset of sweet stimulation and returned to the pre-stimulus level as indicated by red trace (Fig. 1A). The deoxygenated hemoglobin (deoxyHb) level was relatively stable throughout the stimulation period. On the other hand, the oxyHb level increased gradually after the initial transient decrease in response to bitter stimulation, and this increased level was maintained during the recording period (Fig. 1B). The deoxyHb level was again stable. Water stimulation induced a small amount of increase in oxyHb level (Fig. 1C). As shown in Fig. 1D–F, each of the time courses of z-scores calculated for oxyHb concentration corresponds well with each of the raw oxyHB changes.

**Figure 2:**
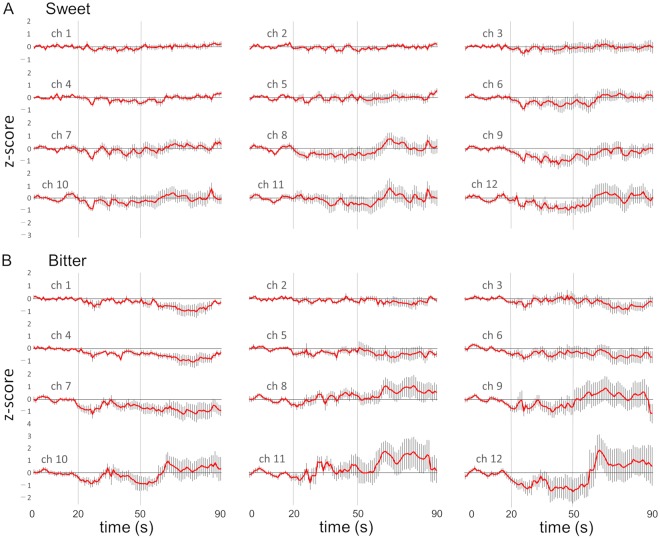
Grand mean z-scores converted from the time series of oxyHb concentration to sweet (**A**) and bitter (**B**) stimulations. The red line represents the mean change in oxyHb (n = 15) and the vertical small line represents the standard error (SE). The vertical bars at 20 s and 50 s represent the onset of stimulation and swallowing, respectively.

However, oxyHb changes were not always consistent, but varied in terms of onset, timing, direction and amplitude among 15 subjects to sweet stimulation and to bitter stimulation (see Supplementary Fig. S1). As a summary of the fNIRS time series data, grand means of z-score are shown for all the channels to sweet and bitter stimuli (Fig. 2). After onset of stimulation, the oxyHb level started to change with larger deflections in chs 10, 11 and 12 than in chs 1, 2 and 3, also after swallowing than before swallowing.

### Stimulation with preferred and disliked food and drink (Experiment 2)

**Figure 3:**
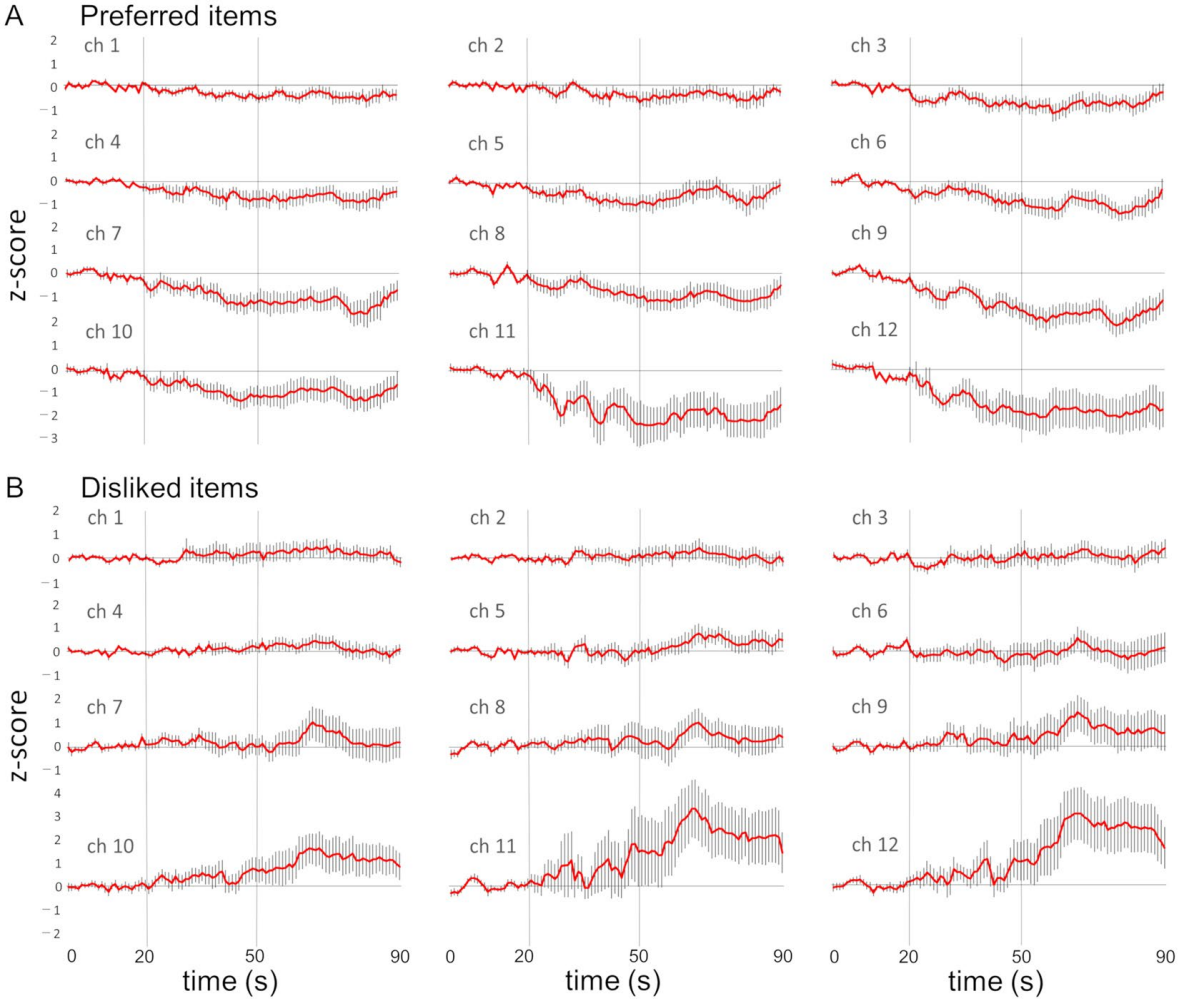
Grand mean z-scores converted from the time series of oxyHb concentration in response to preferred (**A**) and disliked (**B**) items. The red line represents the mean change in oxyHb (n = 20) and the vertical small line represents the SE. The vertical bars at 20 s and 50 s represent the onset of stimulation and swallowing, respectively.

Based on reports on preferred and disliked food or drink, 20 subjects ate or drank the items identified during the recording session. As a summary of the fNIRS time series data, grand means of z-score are shown for all the channels to preferred and disliked items (Fig. 3). Response patterns in each subject in each channel were also variable as shown in Supplementary Fig. S2. The time course of grand means shows a tendency of decrease of oxyHb concentration in response to preferred items and increase to disliked items especially after swallowing.

### Quantitative analyses (Experiments 1 and 2)

**Figure 4:**
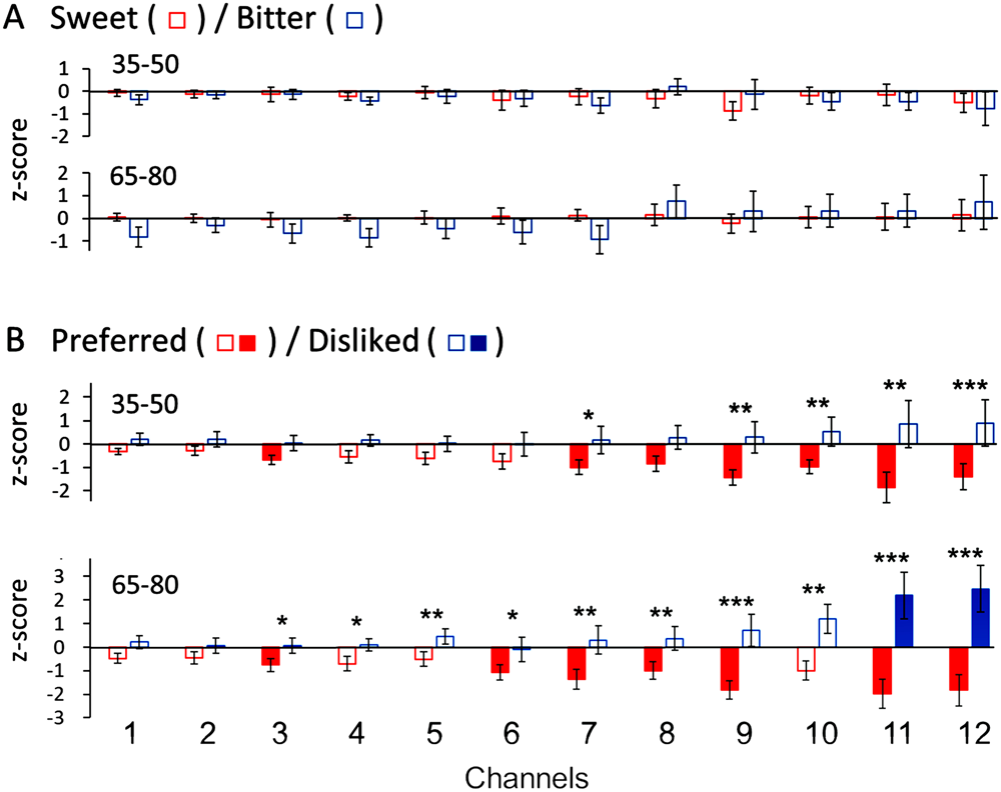
Comparison of mean z-score (±SE) during two time-windows in each channel to stimulations with sweet and bitter solutions (**A**) and with preferred and disliked items (**B**). The two time-windows are 35–50 s after the start of recording (or 15 s before swallowing) and 65–80 s (or the second 15 s after swallowing). Solid bars denote that oxyHb changes were significantly decreased or increased in comparison with the pre-stimulus resting period (*P* < 0.025, *t*-test with Bonferroni correction). Asterisks denote that oxyHb changes between preferred and disliked items are significantly different (two-way ANOVA, Tukey’s HSD test). ^*^P < 0.05, ^**^P < 0.01, ^***^P < 0.001.

We calculated the mean z-score for sweet and bitter stimulations in four time-windows, such as 1) 15 s after onset of stimulation, 2) the following 15 s (or 15 s before swallowing), 3) 15 s after swallowing and 4) the following 15 s. Each mean z-score was compared with that during the pre-stimulus 15 s. The mean z-scores during the second (35–50 s after the start of experiment) and fourth time-windows (65–80 s) are shown in Fig. 4A since large and sustained responses were observed within these time-windows. However, no significant difference was detected compared to the pre-stimulus period in each channel (*t*-test with Bonferroni correction, *P* > 0.025). A two-way ANOVA with taste (sweet and bitter) and channel (from ch1 to ch12) did not detect any significant main effects of solution [*F*(1, 14) = 0.53, *P* > 0.05 for 35–50 s time-window, *F*(1, 14) = 0.77, *P* > 0.05 for 65–80 s time-window] and channel [*F*(11, 154) = 0.14, *P* > 0.05 for 35–50 s, *F*(11, 154) = 1.11, *P* > 0.05 for 65–80 s], indicating no significant difference between the mean z-scores for sweet and bitter stimulations in each channel.

We calculated the mean z-score to preferred and disliked items in the four post-stimulus15-s time-windows and the pre-stimulus period as described above. As shown in Fig. 4B, significant decrease of oxyHb was detected for preferred items in chs 3 and 7–12 during the second time-window (35–50 s) and also in chs 3, 6–9, 11 and 12 during the fourth time-window (65–80 s), while significant increase of oxyHb was found for disliked items only in chs 11 and 12 during the fourth time-window owing to a big intra-subject variation (*t*-test with Bonferroni correction, *P* < 0.025). A two-way ANOVA with taste (preferred and disliked items) and channel (from ch1 to ch12) revealed a significant main effect of solution [*F*(1, 19) = 8.24, *P* < 0.05 for 35–50 s time-window, *F*(1, 19) = 20.18, *P* < 0.001 for 65–80 s time-window] and a significant taste x channel interaction [*F*(11, 209) = 5.76, *P* < 0.001 for 35–50 s, *F*(11, 209) = 10.56, *P* < 0.001 for 65–80 s]. However, the analysis did not detect a significant main effect of channel [*F*(11, 209) = 0.43, *P* > 0.05 for 35–50 s, *F*(11, 209) = 0.87, *P* > 0.05 for 65–80 s]. Significant difference was detected between the mean z-scores for preferred and disliked items in chs 7 and 9–12 during 35–50 s and in chs 3–12 during 65–80 s (post hoc Tukey’s HSD test, see Fig. 4B).

The selection of liquid and solid (or non-liquid) stimuli was unbalanced between preferred and disliked items (see Table 1). When we compared z-scores for ch12 within the time-window 65–80 s between liquid and solid stimuli in both items, the mean ± SD was −1.45 ± 3.10 (n = 8) for preferred liquid and −2.37 ± 2.22 (n = 12) for preferred solid stimuli with no statistically significant difference (*P* = 0.36, two-tailed *t*-test), and 2.83 ± 4.22 (n = 16) for disliked liquid and 1.15 ± 3.34 (n = 4) for disliked solid stimuli with no significant difference (*P* = 0.37). These results suggest that whether the stimuli are liquid or solid has little effects, if any, on the present results, but the hedonic evaluation for these stimuli is more important than their physicochemical properties in changing oxyHb concentrations.

**Table 1:** List of preferred and disliked items for each subject.

Subjects	Preferred	Disliked
P	Berry cheesecake	Bitter solution
Q	Calpis^*^	Scallop
R	Strawberry yogurt	Bitter solution
S	Strawberry shortcake	Spinach
T	Aloe yogurt	Bitter solution
U	Custard pudding	Tomato juice
V	Custard pudding	Bitter solution
W	Orange juice	Hiyashiame^**^
X	Custard pudding	Avocado
Y	Custard pudding	Bitter solution
Z	Custard pudding	Bitter solution
AA	Green tea with milk	Bitter solution
BB	Custard pudding	Banana juice with milk
CC	Custard pudding	Melon juice with milk
DD	Green tea	Bitter solution
EE	Milk tea	Hiyashiame^**^
FF	Milk tea	Hiyashiame^**^
GG	Soufflé cheesecake	Shiitake mushroom
HH	Orange juice	Black tea
II	Soybean milk	Black tea

^*^Lactic acid drink (Japanese brand name: Calpis; Asahi Soft Drinks Co. Ltd., Tokyo); ^**^A traditional Japanese sweet drink made from malt syrup; Bitter solution is 0.03~0.05% sucrose octaacetate.

### Stimulation with four differently flavored jellies (Experiment 3)

Each subject ingested a small amount of four kinds of jellies, containing cream, sucrose, both cream and sucrose or no flavor, randomly during the recording session and simultaneously drew a time-pleasantness curve with a pen on a sheet of paper, to let the subject concentrate on pleasantness evaluation. Soon after each trial, subjects marked a point (or score) on the visual analogue scale (VAS) as an overall pleasantness evaluation. Mean sensory evaluation scores for jellies with only water (no flavor), with cream, with sucrose and with both cream and sucrose are −4.8 ± 3.2, −2.5 ± 4.3, 3.6 ± 2.3 and 5.1 ± 2.3 (mean ± SD, n = 8), respectively. Statistically significant difference was detected between jellies with water or cream and jellies with sucrose or both sucrose and cream (one-way ANOVA, Tukey’s HSD test, *P* < 0.01). There was a tendency that the pleasant jelly with sucrose and cream decreased the oxyHb level, while the unpleasant plain jelly increased the oxyHb level. A sample of oxyHb changes to each of the four jellies is shown in Supplementary Fig. S3.

**Figure 5:**
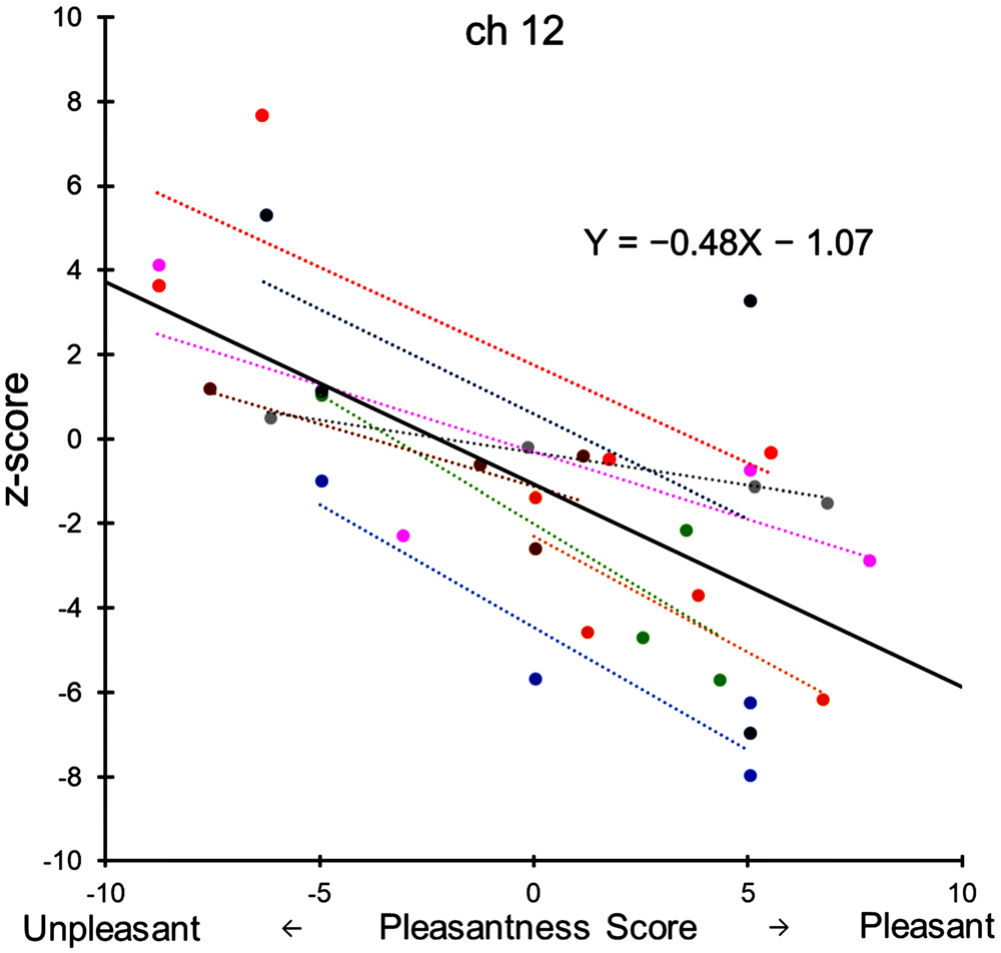
Correlation between pleasantness score and z-score for four differently flavored jellies in eight subjects at channel 12. Each of the subjects showed a good negative correlation (Pearson) between the pleasantness score and the mean z-score (during 15 s after swallowing) as indicated by different colors. The solid regression line was obtained for the total 32 data points using mixed-effects modeling with crossed random effects for subjects and items.

When the relationship between pleasantness scores and mean oxyHb changes (calculated for the first 15 s after swallowing) is shown as a scatter plot diagram for eight subjects in ch 12, each of the subjects showed a good negative correlation indicating that pleasant feeling decreased oxyHb levels, while unpleasant feeling increased the oxyHb level (Fig. 5). A regression line, Y = −0.48 × −1.07, was obtained for the total 32 points using generalized linear mixed-effects modeling with crossed random effects for subjects and items^20^.

**Figure 6:**
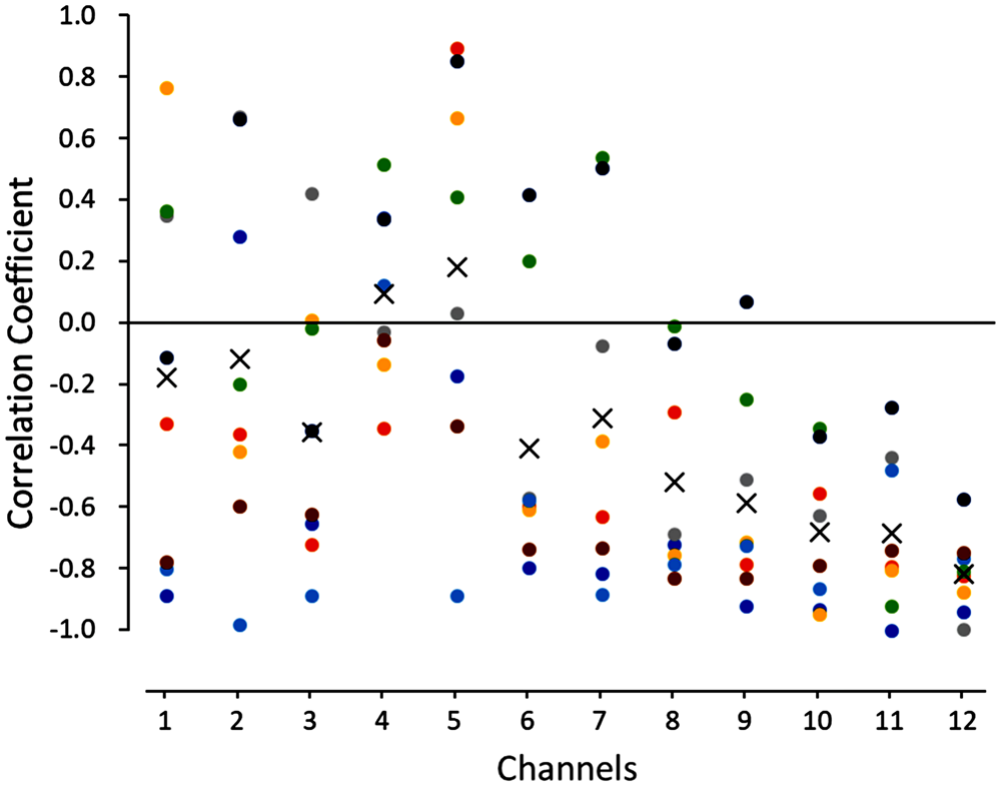
Pearson’s correlation coefficients between pleasant scores and the mean z-scores for oxyHb changes during 15 s after swallowing in each channel for eight subjects. X denotes the mean value in each channel.

Mean correlation coefficients obtained in eight subjects between the pleasant score and oxyHb level are compared across channels (Fig. 6). The values are greatly variable in chs 1–5 (within the dorsal portion of the aPFC), while the values become stable with highly negative correlation toward the ventral direction, and the highest value was obtained in ch 12. One-way ANOVA revealed a significant main effect of channel [*F*(11, 84) = 4.04, *P* < 0.001]. Post hoc Tukey’s HSD test showed that the mean correlation coefficient in chs 9–12 was statistically significantly more negative than that in chs 4 and 5 (*P* < 0.01).

## Discussion

The present study aimed to explore possible reflections of pleasant and unpleasant human emotions in the brain corresponding to the hedonic valence of food and drink. We used a fNIRS technique because it measures hemodynamic responses within the brain in a non-invasive manner and does not require fixation of the head and body. Many previous neuroimaging studies^2,7,8,21–23^ have revealed cognitive and emotional aspects of taste and flavor perception by focusing on the regions such as the frontal/parietal operculum and insula, DLPFC, VLPFC, OFC, cingulate cortex, and amygdala using PET, fMRI and MEG. A key limitation of fNIRS is that it can measure only the lateral surface of the cerebral hemisphere, not the inferior or medial surfaces of the hemispheres or deep brain structures where the above-mentioned taste-related areas exist. We therefore selected the aPFC as a region of interest, because no previous reports have elucidated this region concerning the reward and emotional aspects of taste and flavor. Moreover, the forehead region is more easily accessible for fNIRS measurement than the temporal region, where fNIRS may detect hemoglobin concentration changes in blood vessels to the salivary glands rather than changes within the cerebral cortex^18^.

fNIRS can detect hemodynamic contaminations caused by physiological activities including the systemic circulation and cutaneous blood flow other than cerebral function even in the forehead region. Although we did not monitor systemic circulation in terms of heart rate, mean arterial pressure, and skin temperature, we can cite relevant papers by Kashima *et al*.^24,25^ who used similar taste solutions and commercially available drinks with different palatability as in the present study. They reported that blood pressure was negligibly affected by simple sweet or bitter stimulation and was only slightly elevated by taste and drinks regardless the degree of palatability. Concerning cutaneous blood flow, they found that the blood flow change in the forehead was very small compared to the eyelid, where blood flow increased to sweet, umami and pleasant drinks, and the nose, where blood flow decreased to bitter-tasting stimuli. They also stated that skin blood flow in forehead and cheek were not related to palatability scores. Taken together, our oxyHb changes may have been little contaminated, if any, by systemic circulation and cutaneous blood flow.

The role of the aPFC in the processing of emotional as well as cognitive functions in general is still to be elucidated. Concerning cognitive functions, neuroimaging and electrophysiological studies have shown that the aPFC contributes to several aspects of cognition^19,26,27^, such as the behavior required to concentrate on the current sensory input and the mental processing that accompanies self-generated thoughts^19^, integration of the outcomes of two or more separate cognitive operations^26^, monitoring of action outcomes, selection of alternative tasks in response to goals and decisions to switch tasks^27^, cognitive flexibility and stability^28^, and executive functions^29^. Concerning emotional functions, a recent study^30^ suggested that the lateral portion of the aPFC is involved in emotion, as watching unpleasant pictures was accompanied by an increase in oxyHb, while pleasant pictures were accompanied by a decrease in oxyHb. As discussed below, the present study also suggests that the aPFC reflects pleasant and unpleasant aspects of food and drink, which could be utilized as an objective method for hedonic evaluation of food and drink.

Experiment 1 examined responsiveness to sweet-tasting sucrose and bitter-tasting SOA, which are innately positioned at the extreme opposite from very preferable to very aversive. Since we expected that these innate positions of highly positive and negative hedonics might induce some responses in the aPFC, we allowed subjects to perform passive tasting; in other words, we did not give any particular instruction to the subjects. However, the results varied greatly and we could not obtain any consistent stimulus-dependent responses among subjects. This may be explained partly by the wide variety in sweet-taste preferences^14,31^ and bitter perception^31^ across individuals. Large increase in oxyHb level after swallowing of bitter solution and disliked items may be derived by increased taste intensity due to stimulation of the posterior part of the tongue^32,33^. Furthermore, the aPFC is not the area responsible for direct processing of taste information, but is related to higher-order functions such as cognition, memory, thinking, execution etc. as mentioned above. Under a non-instructed free condition, the subject is unlikely to only think of the taste in the mouth without thinking of anything else during the entire period of the trial. If the aPFC is concerned with the mental processing that accompanies self-generated thoughts^19^, ongoing taste stimuli may have induced various spontaneous thoughts resulting in variability of aPFC activation among subjects. Besides hedonic tone (pleasant or unpleasant) of sweet and bitter solutions, other emotions, such as happiness, sadness, inspiration, depression, liking, wanting and appetite, might occur with different degrees in the subjects, and may also be associated with the hemodynamic changes. Degree of involvement of each component in hemodynamic changes is an important issue to be analyzed in future study.

In Experiment 2, we examined oxyHb levels when the subject ate or drank preferred and disliked edibles that had been prepared specifically for the individual. Although we did not give any particular instruction as in Experiment 1, we detected a tendency for oxyHb levels to decrease during intake of preferred edibles and to increase during intake of disliked edibles, even though preferred and disliked edibles differed among subjects. A significant difference was observed in the ventral portion of the aPFC with no significant difference in laterality. These results suggest that preferred and disliked food and drink elicited stronger hedonic responses than simple sweet and bitter taste stimuli, in turn suggesting that subjects were more hedonically invested in pleasant or unpleasant feelings, resulting in the evocation of hedonic activity changes in the aPFC. Such differences in results between Experiments 1 and 2 might be explained by innate preferences versus learned preferences, as suggested by van den Bosch *et al*.^34^. They examined whether differential neural activation exists between flavor preferences using grapefruit juice as a flavor that was either liked or disliked by subjects and innate taste preference using pleasant sucrose and unpleasant quinine. Although all these stimuli activated regions known to be associated with processing of taste, reward and aversion, such as the OFC, insula, striatum, cingulate cortex and amygdala, they found that flavor preferences that are learned and that have developed through subjective experience are mediated by different neural processes than innate taste preferences. Such findings suggest that in comparison with basic rewards for innate taste preferences, learned preferences for complex stimuli may be regarded as higher-order rewards accompanying a more cognitive evaluation of the flavor. Emotions elicited by likes and dislikes are thus more easily reflected in the aPFC.

In Experiment 3, to more precisely examine the relationship between hedonic evaluations and oxyHb levels from a different perspective, we used four differently flavored jellies: plain jelly with no particular additives; jelly with fresh cream; jelly with sucrose; and jelly with both fresh cream and sucrose. To better allow subjects to pay attention to the hedonics of each jelly, subjects drew time-pleasantness curves on paper during the the trial and evaluated the overall pleasantness on a VAS soon after each trial. The results showed a significant negative correlation between oxyHb content and palatability in the aPFC, particularly within the ventral region, indicating that increased palatability tended to decrease neuronal activity in this region, supporting the results of Experiment 2.

The present paper suggests that a pleasantness-induced oxyHb decrease is displayed in the ventral aPFC. In this respect, several researchers have reported task-induced decreases of regional cerebral blood flow (rCBF) in the aPFC and the dorsal prefrontal cortex using fighting and puzzle video-game tasks^35^, a computer maze task^36^, a driving game task^37^, a reaching task^38^ and goal-directed tasks^39^. Although the reasons for task-related decreases in rCBF have not been fully elucidated, Matsuda and Hiraki^35^ suggested three hypotheses in their video-game task study: 1) the attention demand required for visual stimuli corresponds to inhibition of neural inhibition in the relevant region; 2) decreases in rCBF are caused by blood draining from neighboring areas to active areas, as a passive hemodynamic phenomenon known as ‘vascular steal’; or 3) temporal ischemia, or an ‘initial dip’ phenomenon caused by a rapid increase in neural activity.

Why does oxyHb in the aPFC tend to decrease in response to a pleasant taste? The results from Experiments 2 and 3 suggest that the subjects need to pay attention to or immerse themselves in the pleasantness of taste stimuli without cognitive activities like self-generated thoughts. Hypothesis #1 above may thus be applicable to the present study. Selective attention to the pleasantness and intensity of taste is known to modulate brain activation in gustatory and reward regions^40–43^. For example, Rijn *et al*.^43^ reported that brain activation was higher in the anterior insula and middle and lateral OFC during selective attention to intensity and during selective attention to pleasantness in the right putamen, right ACC and bilateral middle insula. Selective attention to pleasantness in the present study may have activated the above-mentioned areas and information from those areas may be sent to the aPFC.

The possibility of the involvement of vascular steal in the palatability-related oxyHb decrease might be discarded from the following reasons. (1) As shown in Supplementary Figs S1 and S2, channels showing decreased oxyHb (blue) are not always surrounded by channels showing strongly increased oxyHb (red), or increased rCBF, which could steal blood from neighboring areas. Therefore, vascular steal may be negligible within the aPFC. (2) Alternatively, increases in rCBF should be observed outside of the aPFC. The most probable site is the medial mid-anterior orbitofrontal cortex (OFC, BA 11, 13 and 14 according to Kringelbach^6^) which is activated by pleasant taste, flavor and food. Anatomy and cytoarchitectonic maps of human brain and a number of previous imaging studies show that the relevant site within the OFC, which is located on the inferior surface, is apart from our recording site in the aPFC. Therefore, we think that vascular steal by the activation of OFC is less probable in the present study. The ‘initial dip’ hypothesis may not be applicable for the sustained decreases in rCBF in the present study. However, this phenomenon might explain the temporary decrease in oxyHb in response to bitter stimulation (see Fig. 1).

In addition to these explanations, the aPFC may receive emotional inputs from emotion-related brain areas, as neuroanatomical studies have shown that the aPFC has reciprocal connections with the amygdala, cingulate cortex and supramodal cortex in the PFC^26^. It is reported that pleasantness^30^, happiness^44^ and euphoria^45^ reduced rCBF in the frontal cortex. These pleasant emotions are suggested to occur in the case of ingestion of highly palatable food^46^.

Concerning negative valence (unpleasantness) elicited by aversive taste/flavor stimulation, Zald *et al*.^47^ found increased activity to aversive saline solution in the limbic circuits, such as the right amygdala, left anterior orbitofrontal cortex, medial thalamus, pregenual and dorsal anterior cingulate, and the right hippocampus, while Zald and Pardo^48^ showed that exposure to a highly aversive odorant produced strong activities in the bilateral amygdala and in the left orbitofrontal cortex. Small *et al*.^49^ reported that quinine sulfate solution activated hypothalamus, left anterior insular/operculum, left orbitofrontal cortex and anterior cingulate cortex. Concerning emotional depression and/or negative emotions, left amygdala, and related parts of the striatum, pallidum and medial thalamus is involved in unilateral depression^50^. Bilateral activity was shown in the bilateral insula, the bilateral amygdala, parts of the hippocampus, and the right medial orbitofrontal cortex to disgust inducing pictures^51^. The left amygdala was activated to sad and fearful faces more strongly in depressed subjects than healthy control subjects^52,53^ After exposure to bitter-tasting and disliked items, information from these areas for negative valence, emotional depression and negative emotions may be sent directly or indirectly to the ventral portion within the aPFC.

There is a possibility that pleasant and unpleasant emotions modulate activity of the autonomic nervous system (AN), resulting in changes to the systemic circulation such as blood pressure (BP) and pulse rate (PR). Yasui *et al*.^54^. showed that thermotherapy on the neck induced mental relaxation accompanying increased parasympathetic nervous activity and decreased oxyHb concentration in the anterior-dorsal region of the medial prefrontal cortex, suggesting a good correlation between AN activity and cerebral hemodynamics. Although we did not monitor the autonomic activity of subjects in the present study, a similar study in which viewing pleasant and unpleasant pictures decreased and increased oxyHb, respectively, in the aPFC, neither systemic BP nor PR showed significant changes^30^. The present findings that pleasant and unpleasant tastes decreased and increased oxyHb, respectively, within the aPFC may thus be due to activation related to emotion even if activity of the autonomic nervous system is more or less involved. However, since intake of food and drink with different hedonic values is supposed to influence the AN system^55–58^, further study is needed regarding the relationship between hemodynamic changes and AN activity within the aPFC.

A limitation of our study pertains to the number and selection of subjects. The number of subjects was not enough to analyze the results in terms of gender difference and handedness difference. Moreover, we paid little attention to the individual characteristics of taste sensitivity and the degree of emotional response. Future studies should use more subjects with controlled gender and handedness ratio, and results should be analyzed in subjects classified, at least, on the basis of the PROP (6-n-propylthiouracil) test^59^ for taste sensitivity and of the Bermond–Vorst Alexithymia Questionnaire^60^ for evaluation of emotional responses and the levels of alexithymia.

In conclusion, event-related analysis of changes in oxyHb as measured by fNIRS showed that the intake of various taste and flavor stimuli evoking pleasant emotions was accompanied by decreases in oxyHb, while intake of various taste and flavor stimuli evoking unpleasant emotions was accompanied by increases in oxyHb in the ventral portion of the aPFC. These results suggest that hemodynamic changes within this area can be utilized as an effective method for objective evaluation of the hedonic valence of food and drink in addition to subjective evaluation with sensory tests.

## Materials and Methods

### Subjects

A total of 43 healthy volunteer participants were recruited from Kio University, comprising 28 women and 15 men (age range, 21–50 years). Forty subjects were right-handed, and 3 (all women) were left-handed. All participants did not use medications that interfere with taste or were free of sensory, eating, neurological and psychiatric disorders. They were not permitted to eat and drink from 1 hour and to smoke tobacco from 3 hours before the experiment started. Participants were informed about the purpose and safety of the experiments, and written informed consent was obtained prior to participation in the experiment. This study was approved by the ethics committee of the Kio University, and all experiments were conducted according to the Declaration of Helsinki.

### fNIRS recording

**Figure 7:**
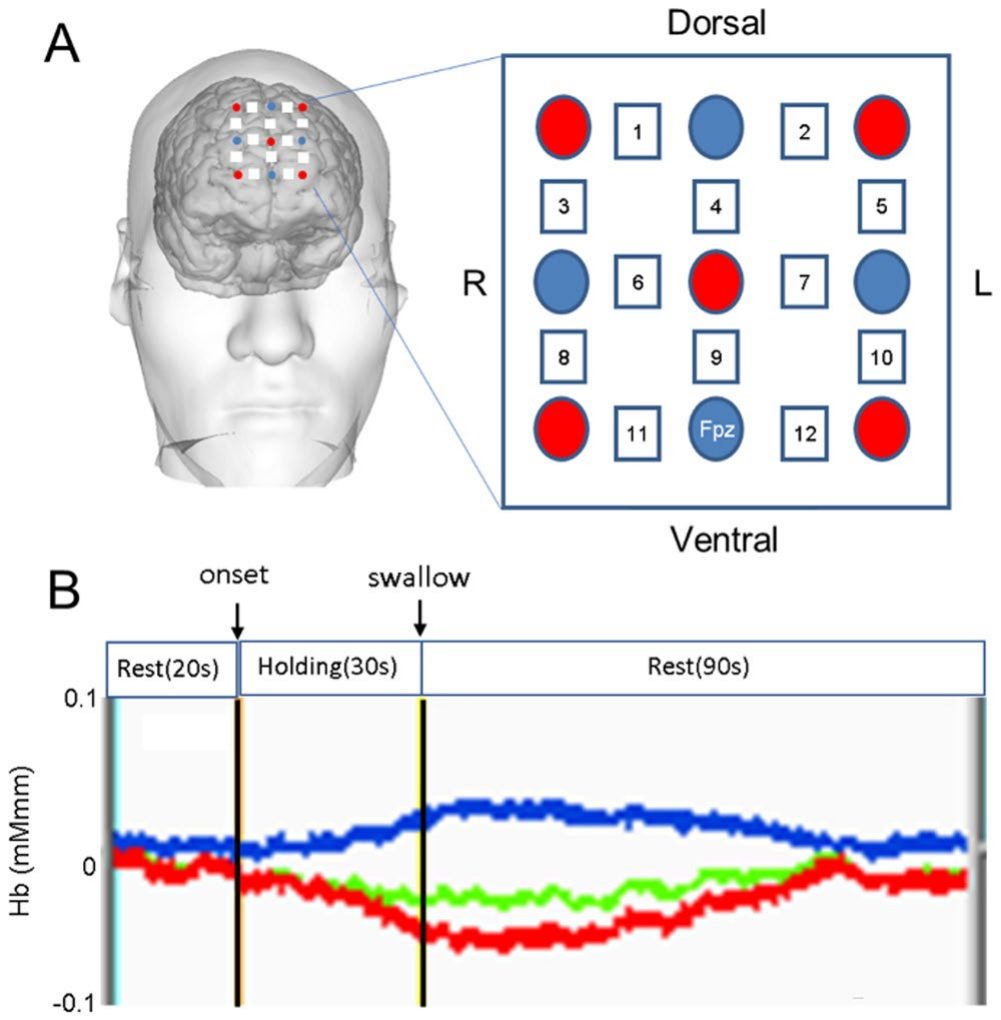
Experimental procedure. (**A**) Locations of optodes and channels. Red circle, emitter; blue circle, detector; square, channel with number, Fpz, a point defined in accordance with the International 10/20 system used in electroencephalography. The distance between emitters and detectors was set at 3 cm. The left side of the figure shows the cortical location of channels on a rendered brain. (**B**) Experimental design with a sample recording. Red trace, oxyHb level; blue trace, deoxyHb level; green trace, totalHb level.

We employed a fNIRS system (FOIRE-3000; Shimadzu, Kyoto, Japan) using continuous-wave laser diodes with wavelengths of 780, 805 and 830 nm. Using these three types of near-infrared light, we detected concentrations (mMmm) of oxygenated hemoglobin (oxyHb), deoxygenated hemoglobin (deoxyHb) and their sum (totalHb) by applying a modification of the Beer-Lambert law^61^ in 12 channels at a sampling rate of 12 Hz. We used 9 optodes (3 × 3 arrays), comprising 5 light emitters and 4 detectors (Fig. 7A). Optodes were kept in place with a soft plastic holder. The distance between emitters and detectors was set at 3 cm: each pair of adjacent emitting and detecting optodes defined a single measurement channel, which allowed for the measurement of oxyHb and deoxyHb changes in 12 channels (Fig. 7A).

The probe matrix consisting of 9 optodes was placed on the subject’s forehead with the center of the lowest array placed on Fpz in accordance with the International 10/20 system used in electroencephalography. In this manner, the probe matrix was positioned to record brain activities at a depth of approximately 3 cm within the aPFC.

### Procedure

#### Stimulation with sweet and bitter solutions (Experiment 1)

Fifteen subjects (10 women, 5 men; age range, 21–50 years) were recruited. Thirteen were right-handed, and two women were left-handed. The subject sat on a chair in a relaxed state in a quiet room at 25 °C with relative humidity around 50%. During the test session, each subject kept a straw between the upper and lower lips and after a 10-s resting period received an aliquot (6 ml) of distilled water, sucrose solution (10%) as a sweet stimulus or sucrose octaacetate (SOA, 0.03% or 0.05%) as a bitter stimulus. The liquid was administered into the mouth by a syringe positioned at the other end of the straw. An experimenter pushed the piston of the syringe at a constant rate to deliver the liquid within a few seconds, then the straw was gently withdrawn from the mouth. As the time schedule for one trial, the subject waited for 20 s, received one of the liquids and held it in the mouth for 30 s, then swallowed following a signal from the experimenter and kept quiet for another 90 s (Fig. 7B). The subject then rinsed the mouth to remove the remaining taste. This trial was repeated 2 or 3 times for each stimulus in a randomized manner, with a rest period of about five min provided between each trial. During the trial, the subject was asked to look at a marker placed on the front wall and to restrain from head movements.

#### Stimulation with preferred (hedonically positive) and disliked (hedonically negative) food and drink (Experiment 2)

Before the experiment, candidates were asked to nominate their most preferred and disliked food or drink. We selected 20 subjects (12 women, 8 men age range, 21–24 years) with distinct likes and dislikes. Nineteen subjects were right-handed and one woman was left-handed. The time schedule for one trial was also 20 s of rest, 30 s of intraoral stimulus-holding with slight chewing for non-liquid stimuli until swallowing, then another 90 s of rest. No particular instruction was given except for an instruction to refrain from head or body movements despite any pleasant or unpleasant feelings.

Food items used as preferred and disliked food and drink for each subject are listed in Table 1. For subjects who disliked any foods or drinks that have bitter taste, we used bitter aqueous solution (0.03~0.05% SOA). Liquids were administered into the mouth using the same method as described above. Soft materials, such as soufflé cheesecake, custard pudding, scallops boiled in water and avocado, were delivered into the mouth with a teaspoon. The subject was asked to move the jaw slowly and mash between the tongue and palate. Spinach was prepared as boiled spinach seasoned with soy sauce and a teaspoonful of the leaves cut into small pieces was put into the mouth. The subject chewed them slowly and gently, then swallowed. Shiitake mushroom, boiled, seasoned and cut into small pieces, was put into the mouth with a teaspoon. The subject chewed them slowly and gently, then swallowed. A couple of experimenters carefully monitored the subjects’ behavior and movements including the mouth, head and body throughout the recording session. Recordings interfered with movement artifacts or crunching noise could be easily detected by watching the fNIRS responses on the monitor display and discarded immediately when detected during fNIRS recording. More precisely, if oxyHb, deoxyHb and total Hb levels abruptly shifted synchronously in more than 1/3 of the channels corresponding to the observed motion, we discarded the recording. The trial was repeated until we obtained good recording with negligible artifacts. If the similar phenomena were found during later off-line analysis, we also removed the data.

#### Stimulation with jellies with 4 different tastes (Experiment 3)

To examine the relationship between hemodynamic changes and sensory evaluation, we administered the following four different flavored jellies to eight subjects (six women, two men; age range, 21–26 years). All subjects were right-handed. Commercially available gelatin powder (2 g) was dissolved in 100 ml of the following four liquids by heating, dispensed every 5 ml and cooled and solidified: 1) water; 2) 15% sucrose; 3) 40% fresh cream; and 4) a mixture containing both 15% sucrose and 40% fresh cream. An aliquot (about 5 g) of each of these 4 jellies was placed into the mouth of each subject 2 times randomly. The time schedule for one trial was 10 s of rest, 20 s of intraoral stimulus-holding with slight chewing until swallowing, then another 30 s of rest.

As a sensory test to evaluate the pleasantness of each jelly, each subject drew a time-pleasantness curve after administration of the stimulus on a sheet of paper with a pencil, rating from the vertical 0 (neutral) level and drawing upward depending on the pleasantness of the stimulus up to the maximum score of +10 (very pleasant) or downward to a minimum of −10 (very unpleasant), while simultaneously moving the pencil from the leftmost horizontal 0 point (start of recording) to the rightmost end of the trial. The purpose of this drawing is to get the subject to concentrate on the pleasantness of the stimulus. Soon after each trial, the subject made an overall evaluation on a visual analogue scale (VAS) to evaluate the level of pleasantness. The VAS consisted of a horizontal line with one anchor point at each extreme. The descriptions “very unpleasant” and very pleasant were placed at the left and right ends of the scale, respectively. The VAS was scored from −10 to +10, with 0 (neutral) in the center.

#### Data analysis

We used oxyHb levels as a marker of cortical activity, because oxyHb was the most sensitive indicator of changes in regional cerebral blood flow (rCBF) and provided the strongest correlation with the BOLD (blood-oxygen-level-dependent) signal among the three fNIRS parameters monitored^62–64^.

We removed trials from analysis if the trial included movement artifacts as described above. After averaging raw data for each stimulus and water, the water response was subtracted from each taste response to eliminate responses possibly due to intra-oral somesthetic stimulation.

As for data processing, the magnitude of oxyHb concentration was sampled every 85 ms and ten data points were averaged for smoothing, providing data every 0.85 s, and z-scores were calculated as the difference in the means of the pre-stimulus baseline and taste condition divided by the standard deviation (SD) of the baseline:

z-score = (mean taste condition – mean baseline) / SD of the baseline

Although hemodynamic data of fNIRS were relative values and could not be averaged directly across channels or subjects, the z-score, as normalized data, could be averaged regardless of the unit^35,65^.

Grand means of oxyHb changes for the subjects in each channel were obtained, and the mean z-score in the pre-stimulus resting period was compared with that in each 15 s time-window after the start of taste stimulation by using two-tailed *t*-test with Bonferroni correction. To compare z-scores between sweet and bitter stimulations (Experiment 1) and preferred and disliked items (Experiment 2) in each channel, a two-way analysis of variance (ANOVA) followed by post hoc Tukey’s honestly significant difference (HSD) test was used. To determine the association between the pleasantness evaluation and oxyHb levels, Pearson’s correlation coefficients were calculated between VAS ratings and z-scores within each channel. To compare the mean correlation coefficients across channels, a one-way ANOVA followed by post hoc Tukey’s HSD test was used. Values of *P* < 0.05 were considered statistically significant except for Bonferroni correction. Statistical analyses were performed using a software program (IBM SPSS Statistics, ver. 25). For mixed-effects modeling, the lme4 package in the R statistics platform (ver. 3.4.0, R foundation for Statistical Computing, http://www.r-project.org; R Core Team) was used.

## Electronic supplementary material

Supplementary Information
