# Supplementary material for: Activity of frontal pole cortex reflecting hedonic tone of food and drink: fNIRS study in humans
Source: Sci Rep. 2018 Nov 1;8:16197. doi: 10.1038/s41598-018-34690-3 (PMC6212539; doi:10.1038/s41598-018-34690-3)
Supplement: Supplementary file 1 — Supplementary Information [file 41598_2018_34690_MOESM1_ESM.docx]

Supplementary Information

Activity of frontal pole cortex reflecting hedonic tone of food and drink: fNIRS study in humans

Yuji Minematsu, Kayoko Ueji and Takashi Yamamoto

Supplementary Figures S1, S2 and S3

Legend for Supplementary Figure S1

Summary of z-scores in each channel across 15 subjects (A to O) to sweet (sucrose) and bitter (SOA) stimulations in the upper and lower panels, respectively. Z-scores were obtained for oxyHb responses during 70 s period (30 s before swallowing + 40 s after swallowing) after stimulus onset vs. 20 s pre-stimulus baseline. Subjects are arranged in order of the smallest to the largest mean z-scores in response to sweet stimulation, from left to right. When z-scores were compared for each channel between sweet and bitter stimulations across 15 subjects, no significant difference was detected (*P* > 0.05, paired *t*-test), as indicated in the rightmost column. However, comparison of mean z-scores between sweet and bitter stimulations in each subject revealed that 8 subjects (53.3%) showed a significant difference (*P* < 0.05 or 0.01, paired *t*-test) between the two stimuli, as indicated below the lower panel.

Channels showing negative z-scores are indicated in 4 ranks of blue color (weak, z-score < 0; medium, z-score < -1; strong, z-score < -2; strongest, z-score < -3) and channels showing positive z-scores are shown in 4 ranks of red color (weak, z-score > 0; medium, z-score > 1; strong, z-score > 2; strongest, z-score > 3). * *P* < 0.05, ** *P* < 0.01, *** *P* < 0.001.

**Supplementary Figure S1**


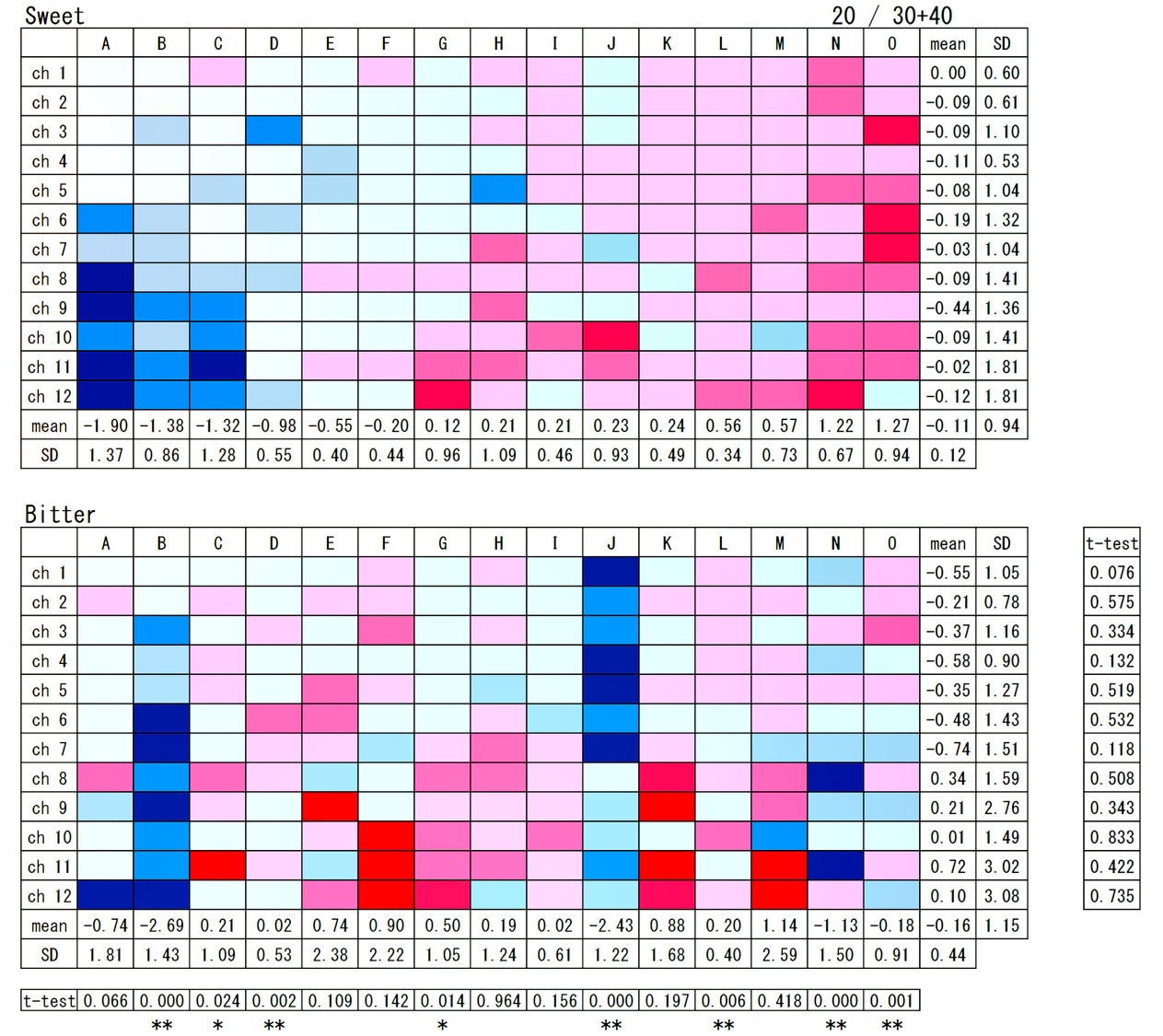


Legend for Supplementary Figure S2

Summary of z-scores in each channel across 20 subjects (P to II) in response to preferred and disliked items in the upper and lower panels, respectively. Z-scores were obtained for oxyHb responses during 70 s period after stimulus onset vs. 20 s pre-stimulus baseline. Subjects are arranged in order of the smallest to the largest mean z-scores in response to preferred edibles, from left to right. Difference in z-scores between preferred and disliked stimuli in each subject is shown as the *P* value of a paired *t*-test (two-tailed, n = 12) under the panel. Similarly, difference in z-scores between preferred and disliked items in each channel is shown in the rightmost column of the panel (*t*-test, n = 20). The most important finding is that the mean z-score for preferred items was negative in 18 among 20 subjects (90 %), while for disliked items in only 7 (35 %), and was smaller than that for disliked items with two exceptions (subjects U and II). Comparison of the mean z-scores between preferred and disliked items in each subject revealed that 17 of the 20 subjects (85 %) showed a significant difference (*P* < 0.05, 0.01 or 0.001, paired *t*-test), as indicated below the lower panel. When mean z-score in each channel was compared between preferred and disliked items across 20 subjects, a significant difference (*P* < 0.05 or 0.01, paired *t*-test) was detected in 8 channels (chs 4, 5, 7-12) as indicated in the rightmost column. See the legend to Supplementary Figure S1 for further explanation of the diagram.

**Supplementary Figure S2**


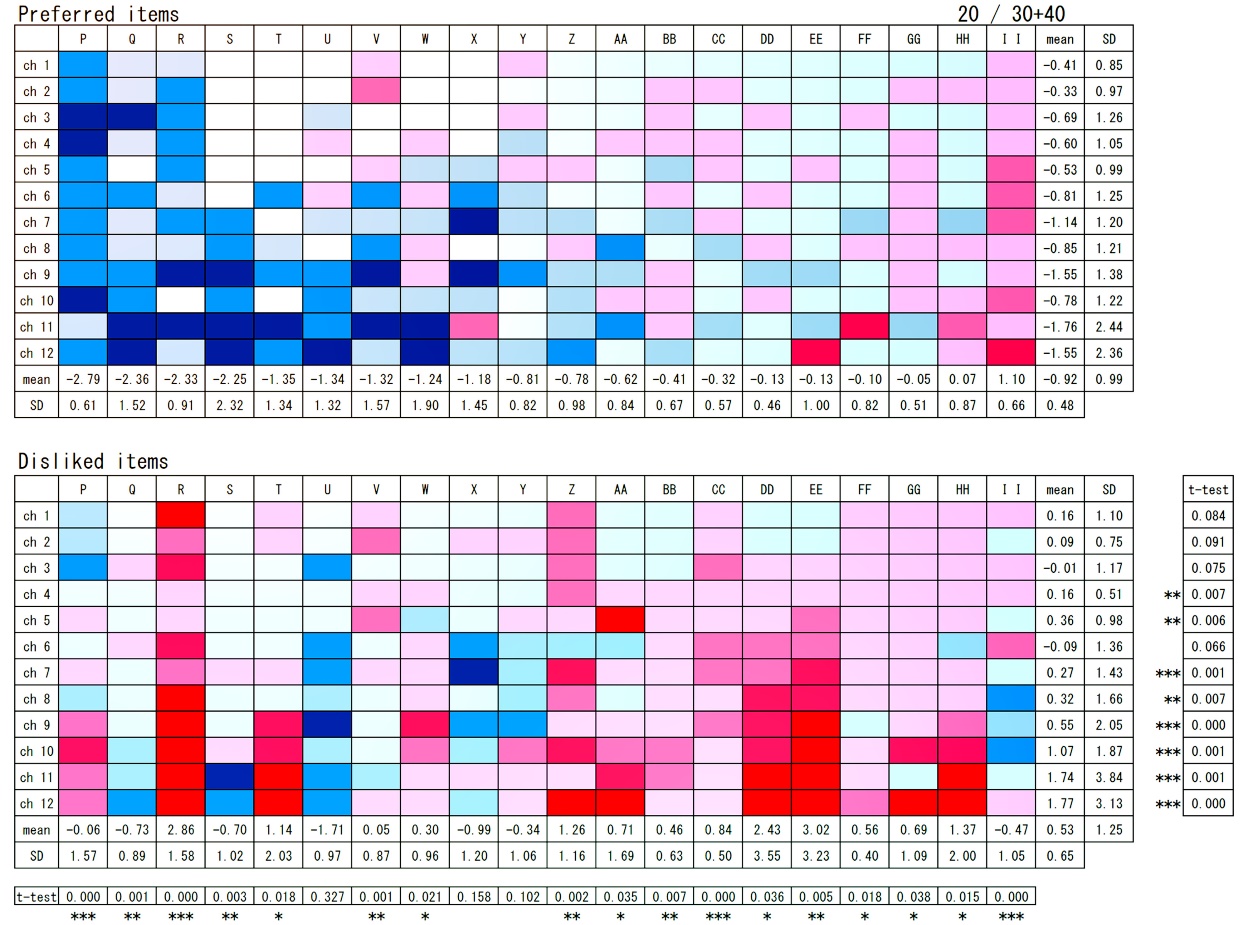


**Supplementary Figure S3**


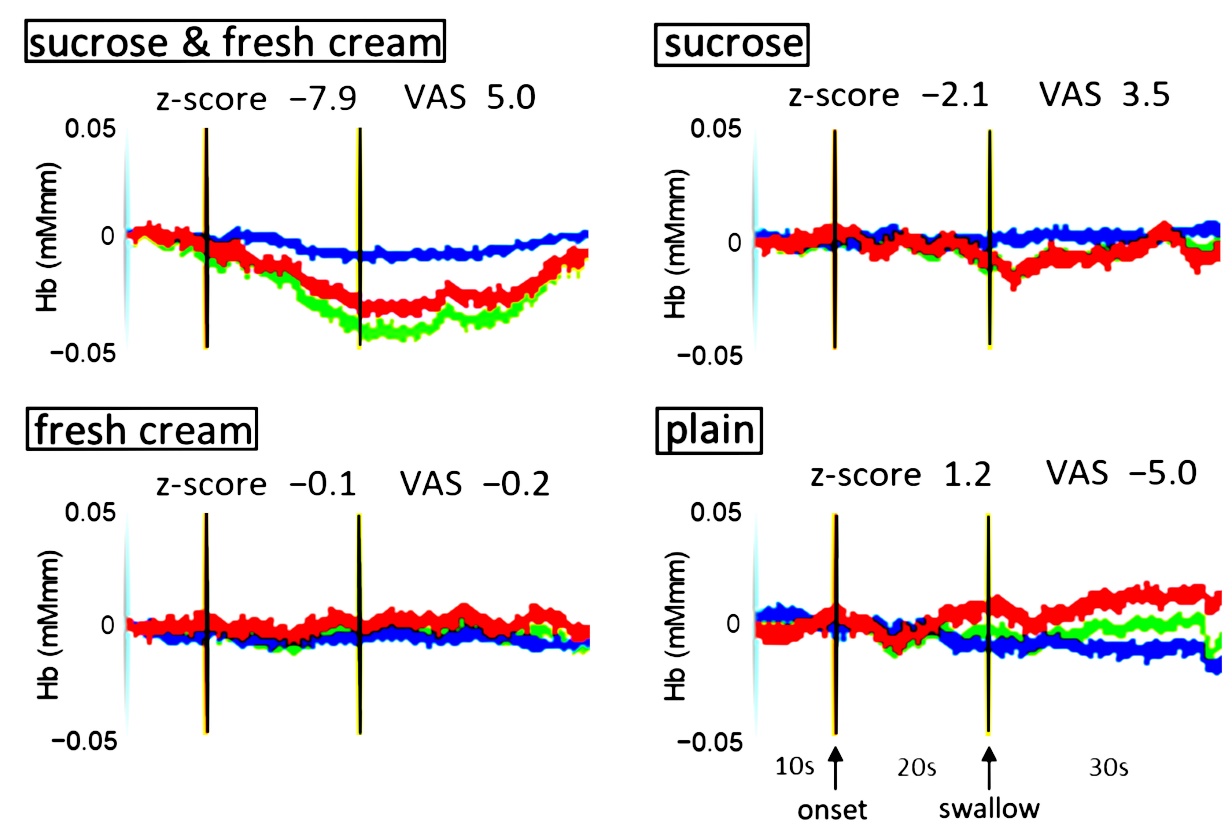


Legend for Supplementary Figure 3.

A sample of hemodynamic changes to four kinds of jellies. The records were obtained from ch 12 in a subject. OxyHb level decreased when the subject felt pleasant to the jelly with the flavor of sucrose and fresh cream, but increased to unpleasant plain jelly as indicated by z-score and visual analogue scale (VAS) rating.
